# Pseudogene-derived lncRNAs: emerging regulators of gene expression

**DOI:** 10.3389/fgene.2014.00476

**Published:** 2015-02-04

**Authors:** Michael J. Milligan, Leonard Lipovich

**Affiliations:** Center for Molecular Medicine and Genetics, Wayne State University School of Medicine, Detroit, MI, USA

**Keywords:** lncRNA, pseudogenes, genome wide, regulation of gene expression, ncRNA, transcription, genetic

## Abstract

In the more than one decade since the completion of the Human Genome Project, the prevalence of non-protein-coding functional elements in the human genome has emerged as a key revelation in post-genomic biology. Highlighted by the ENCODE (Encyclopedia of DNA Elements) and FANTOM (Functional Annotation of Mammals) consortia, these elements include tens of thousands of pseudogenes, as well as comparably numerous long non-coding RNA (lncRNA) genes. Pseudogene transcription and function remain insufficiently understood. However, the field is of great importance for human disease due to the high sequence similarity between pseudogenes and their parental protein-coding genes, which generates the potential for sequence-specific regulation. Recent case studies have established essential and coordinated roles of both pseudogenes and lncRNAs in development and disease in metazoan systems, including functional impacts of lncRNA transcription at pseudogene loci on the regulation of the pseudogenes’ parental genes. This review synthesizes the nascent evidence for regulatory modalities jointly exerted by lncRNAs and pseudogenes in human disease, and for recent evolutionary origins of these systems.

## REDEFINING THE HUMAN GENE COUNT

Classical definitions of genes focus on heritable sequences of nucleic acids which can encode a protein (White et al., 1994). The question of how many genes the human genome contains has been an evolving point of contention since before the Human Genome Project. In 1994, the estimated total human protein-coding gene count was 64,000–71,000 genes (White et al., 1994). The higher gene estimate was based on partial genome sequencing, GC content, and genome size. The lower bound of 64,000 took into account expressed sequence tags (ESTs) and CpG islands as additional prediction factors. In 2000, a new count of actively transcribed genes was estimated at 120,000 using the TIGR Gene Index, based on ESTs, with the results from the Chromosome 22 Sequencing Consortium (Liang et al., 2000). 1 year later, Celera arrived at only 26,500–38,600 protein-coding genes using their completed human genome and comparative mouse genomics (Venter et al., 2001). The Human Genome Project, which used tiling-path sequencing as opposed to Celera’s shotgun sequencing, converged on a similar estimate (Lander et al., 2001).

Following the sequencing of the human genome, focus has shifted toward understanding gene function. In 2005, the FANTOM (Functional Annotation of Mammals) Consortium determined that the mouse genome harbored more non-coding genes than coding genes (Carninci and Hayashizaki, 2007). In a parallel project to FANTOM, the ENCODE (Encyclopedia of DNA Elements) Consortium began exhaustively surveyed the epigenetics and regulation of the whole genome (Birney et al., 2007; Consortium ENCODE Project, 2012). ENCODE’s continuing effort to recount human genes (GENCODE) using the study of genetic landmarks indicative of transcription and next generation sequencing has allowed them to arrive at a current total of just under 58,000 genes as of 2013 (gencodegenes.org). Of these 58,000 genes ENCODE only defines approximately 20,000 genes as coding, with almost all of the other genes being classified as pseudogenes and non-coding RNA (ncRNA). Early studies of the mouse transcriptome by the FANTOM Consortium first motivated the redefinition of a gene into a transcriptional unit as a consequence of large numbers of lncRNA genes discovered (Carninci et al., 2005). Subsequently, the expansion of known metazoan lncRNA repertoires (Derrien et al., 2012; Necsulea et al., 2014) has invigorated the perception that lncRNAs are omnipresent, although many lncRNAs are expressed at low levels, in a more tissue-specific fashion, and with greater intertissue variability relative to protein-coding genes (Derrien et al., 2012). The fact that non-coding genes are so ubiquitous makes it reasonable to hypothesize that their ncRNA products may be extensively involved in the regulation of protein-coding genes. In fact, evidence in favor of specific lncRNAs’ regulatory inputs into particular protein-coding genes is emerging, as the subsequent section will detail.

## LONG NON-CODING RNA: STRUCTURE, IDENTIFICATION, AND FUNCTION

Non-coding RNA (ncRNA) is a broad definition which encompass all types of RNA that lack empirical evidence of translation into protein. NcRNA is identified bioinformatically by: the absence of open reading frames in an RNA, size selection, and low potential product homologies to known proteins. To further classify ncRNA, a biologically arbitrary 200 nt threshold is used to distinguish long non-coding RNA (lncRNA), as transcripts that lack >100 aa ORFs, from short ncRNAs with known functions, most of which are <200 nt (Dinger et al., 2008). This lncRNA length cutoff is hence exclusionary as it defines lncRNAs as RNAs above short-ncRNA length that lack mRNA properties. The HUGO Gene Nomenclature Committee has approved the utilization of this limit (Wright, 2014). As lncRNA functional mechanisms become better understood, a biologically relevant classification method will likely replace this length threshold. The simplest criterion for translation prediction is a threshold of 300 nucleotides (Okazaki et al., 2002), which is used because 88% of protein-coding genes have a 100 aa long or longer protein product (Frith et al., 2006). Using randomly generated transcripts, a 300 nt threshold will classify many non-coding ORFs as protein-coding (Dinger et al., 2008). LncRNAs may require additional criteria to categorize them: high rates of evolutionary substitution, and low similarity to known protein domains are useful in this regard (Dinger et al., 2008). Certain short ORFs of lncRNAs are recurrently translated into peptides whose cellular localization may be indicative of function (Slavoff et al., 2013), although only targeted mutagenesis (for example, genome editing) to abrogate the lncRNA-encoded short ORFs would prove conclusively whether these ORFs are relevant to the lncRNAs’ functions. New experimental methods are beginning to address lncRNA-translation functionality. Ribosomes are used in a technique called Ribosome Profiling followed by sequencing (Ribo-Seq) as a shield for RNA from RNase yielding indirect evidence of translation (Ingolia et al., 2009). RNA fragments derived from this method may also include those protected by non-ribosomal proteins, resulting in false-positive lncRNA translation assessments and hence necessitating filtration. Ribosome Profiling separates coding from non-coding regions, but fails to differentiate some lncRNA from untranslated regions (UTRs) and other types of ncRNA (Guttman et al., 2013). Since Ribosome Profiling does not prove translation, mass spectrometry should be used to verify translation (Ingolia, 2014). The finding that only a minority of lncRNAs are associated with ribosomes (Guttman et al., 2013) is consistent with the earlier report that implicated only a small subset of human lncRNAs in persistent translation based on direct mass spectrometric evidence without ribosome profiling (Banfai et al., 2012).

Methods to assess and quantitate lncRNA expression include: cDNA library construction and sequencing, RNA-seq, cap analysis of gene expression (CAGE) and poly(A)-position profiling by sequencing (3P-Seq; Jan et al., 2011; Fort et al., 2014). CAGE is a method used to determine RNA expression profiles using 5’ ends of RNA molecules, and 3P-Seq is another method of RNA profiling which relies on identifying polyadenylated RNA termini (Takahashi et al., 2012). The functional roles of non-coding RNA are heterogeneous, including both upregulation and downregulation of gene expression. LncRNA is remarkable for its functional heterogeneity, relative to miRNA, which functions mainly as a post-transcriptional suppressor. LncRNAs have many mechanisms by which they regulate cell cycle progression, apoptosis, and differentiation (Rossi and Antonangeli, 2014) and are essential for numerous processes, such as erythrocyte differentiation (Alvarez-Dominguez et al., 2014). Perhaps the best-studied function of lncRNA involves inactivating one of the two X chromosomes in female placental mammals. The lncRNA Xist binds to the X chromosome and recruits silencing factors that propagate the epigenetic landscape. Several well-characterized lncRNAs act as scaffolding for methyltransferases, polycomb proteins, and other epigenetic modifiers: Xist, which spreads PRC2-dependent silencing (Lee et al., 1999; Kung et al., 2013; Simon et al., 2013), and the similarly PRC2-modulating HOTAIR (Kogo et al., 2011), as well as transcripts interacting with other proteins such a WDR5 for chromatin remodeling (Wang et al., 2011).

As more lncRNA mechanisms are elucidated, several themes for lncRNA action are emerging. In the nucleus, lncRNAs commonly bind chromatin and chromatin modifying proteins, facilitating epigenetic regulation (Hawkins and Morris, 2010). Specific lncRNAs also bind certain transcription factors. This either creates a tether between the transcription factor and a gene, or sequesters the transcription factor by acting as a decoy. LncRNAs may also act by influencing subcellular localization of splicing factors, or even disrupting polymerase activity. In the cytoplasm, lncRNA can act by: binding miRNA directly, binding a miRNA’s target sequence, modifying mRNA stability, preventing transcription factors from entering the nucleus, or binding protein complexes which regulate cell proliferation and death (Kung et al., 2013). However, the vast majority of individual lncRNA mechanisms remain unknown.

## PSEUDOGENE STRUCTURE AND FUNCTION

Pseudogenes are copies of protein-coding genes that are thought to no longer produce the same functional product as their parental gene, but still share a high sequence similarity and can therefore regulate their parental genes through the generation of lncRNAs. They can lose the ability to function in the same way as their parental gene by truncation or mutation relative to the parental gene. When further describing pseudogenes they can be divided into two major classes: processed and unprocessed pseudogenes (Li et al., 2013). Unitary pseudogenes are a rare subclass of unprocessed pseudogenes, which have diverged to the point that they no longer have an identifiable parental gene in the genome in which they reside. Unprocessed pseudogenes can be generated by segmental duplications and then disabled by one or more mutations. They typically will have a promoter, introns, and exons. Over time, however, these elements are likely to lose their function. This can happen for newly generated unprocessed pseudogenes for two reasons: either a lack of selective pressure to retain the existing nucleotide sequence or ORF (because the gene no longer serves a relevant biological function), which allows the gene to evolve at the prevailing neutral rate, or selective pressure against the retention of the protein-coding capacity, because of a detrimental effect associated with increased protein concentration or a dominant negative effect arising from the changed protein sequence. The second major class of pseudogenes are processed pseudogenes, which are derived from the reverse transcription of a parental gene’s mRNA. They therefore are limited in structure to a single exon.

**FIGURE 1:**
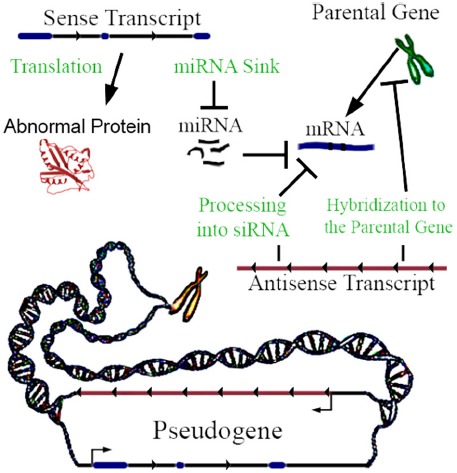
**Pseudogene functional mechanisms.** In this example, a three exon hypothetical pseudogene yields a sense and antisense transcript. The few known examples of antisense transcription of pseudogenes clearly highlight the repression of parental genes. However, hypothetically activating relationships are also possible.

Not all pseudogenes are actively transcribed, but when transcribed they can be functional. Lethe is an example of a pseudogene that produces lncRNA which binds RelA, inhibiting RelA’s ability to bind NF-κB gene promoters (Rapicavoli et al., 2013). It has been traditionally assumed that most pseudogenes are not translated into proteins, because they are claimed to not yield functional mRNAs (Lodish, 2013); this assumption has recently been challenged. Although no comprehensive mass spectrometric and RiboSeq assessment of this fact exists to date, it has been shown using mass spectrometry in mice that a small subset of pseudogenes are translated into proteins (Brosch et al., 2011). For pseudogenes that are transcribed, a key mechanism is interaction with machinery regulating the parental gene’s expression (Li et al., 2013). Pseudogene transcripts act in four ways to regulate gene function (Figure 1), and can be differentially expressed between 0.03- and 45-fold in proliferating versus senescent human cells, which suggests function (Abdelmohsen et al., 2013). Regulation may be enacted through miRNA hybridization to the pseudogene’s sense transcript; in this way, the transcript may act as a sink of miRNA. Sense pseudogene expression at approximately 1% of the parental gene mRNA level can have a significant dose-dependent effect on parental gene transcript concentration and on other gene targets by acting as a miRNA sink (Supplementary Figure 3a of Poliseno et al., 2010). In some cases, genes may require high levels of expression to alter miRNA concentration to an observable degree by acting as a miRNA sink (Denzler et al., 2014). However, miRNA mimics suggest that even minute changes in miRNA concentration may be amplified through influencing histone modifications at gene promoter sites (Younger and Corey, 2011). Non-miRNA mechanisms of sense pseudogene transcription include non-coding ABCC6 pseudogene transcripts, which impact ABCC6 parental gene mRNA level (Piehler et al., 2008), and the activation of the MAP kinase pathway by the BRAF pseudogene sense RNA transcription (Zou et al., 2009). When a pseudogene is transcribed in antisense relative to its parental gene, its RNA can hybridize to the parental gene’s RNA, or epigenetically target the promoter of the parental gene (Johnsson et al., 2013). SiRNA can be generated by processing pseudogene transcripts, which can silence genes by interfering with their transcription (Watanabe et al., 2008). Finally, a pseudogene can be translated into a truncated or mutated protein with novel functionality (McEntee et al., 2011). In addition to these mechanisms, transcribed pseudogenes—which yield lncRNAs—can, in theory, also function by any lncRNA mechanisms, and/or, through short-RNA biogenesis, may be able to regulate diverse genomic loci in addition to the cognate parental genes. Recently, Gencode has developed a distinct and hierarchical set of biotypes describing pseudogenes and differentiating them from protein-coding genes (Pei et al., 2012). Notably, PTENP1 copy number losses are be associated with colon-cancer, and differential expression of it’s parental gene (PTEN; Poliseno et al., 2010).

## CONCURRENT RECENT EVOLUTION OF lncRNAs AND PSEUDOGENES

### LNCRNA EVOLUTION: LIMITED CONSERVATION

Although the earliest-described functional lncRNAs tended to be well-conserved, the breadth of recent lncRNAome catalogs has empowered the realization that at least one-third (in an early ENCODE Consortium estimate), and likely more than one half (in more recent studies), of human lncRNAs are not well conserved across non-primate mammals, and that up to 20% of human lncRNAs may be hominid-specific, very large percentages relative to protein-coding regions (Derrien et al., 2012; Necsulea et al., 2014; Washietl et al., 2014). One plausible explanation for this lack of conservation is that most lncRNAs are non-adaptive, but are instead exaptive. This refers to a change in DNA which doesn’t necessarily serve an adaptive purpose, but may later gain a new adaptive function through selection. For instance, point mutations may create splice sites (GT, AG) and/or polyadenylation signals (AATAA, ATTAAA) where there were previously none; if a repetitive element containing a weak promoter inserts upstream, a new transcriptional unit is born, which is transcribed as an lncRNA. The stochastically originating transcript does not owe its existence to selection, but can become a substrate on which selection acts. These events exemplify an evolutionary mechanism for gene birth and death that generates transcribed substrates for selection, and hence for adaptive evolution (Gilbert et al., 1997). Therefore, transcribed sequences may be reservoirs for genetic material and are not disposable even when adaptation doesn’t govern their evolution (Brosius and Gould, 1992). Approximately half of mouse lncRNAs are not in rat (and vice versa) and, while a result of this gene gain and loss process, still contribute to lineage-specific gene expression (Kutter et al., 2012). While no systematic genomewide analysis of exaptation as a contributing cause of interspecies lncRNA gene repertoire diversity has been conducted, exaptation has influenced regulatory element (Franchini et al., 2011) and gene (Wissler et al., 2013) origins in mammals and in a socially complex insect order, respectively. Exaptation-driven origin of a novel lncRNA family in primates has been traced to the retroposition of an endogenous antisense mRNA transcript as well (Schmieder et al., 2008). The latter lacks a known function but its brain expression and high-complexity splicing are consistent with functionality.

A contrasting viewpoint stipulates that non-conservation of lncRNA gene bodies does not imply a lack of function (Morris and Mattick, 2014). Some well-conserved aspects of lncRNA genes are RNA secondary structures and gene promoter sequences (Johnsson et al., 2014). Once a new lncRNA becomes beneficial in regulation, it will fall under functional constraint. LncRNA exons identified by trimethylation at H3K4/H3K36 and absence of known protein products show enhanced conservation versus intergenic regions and reduced conservation versus known protein-coding exons; this level of conservation makes secondary structure conservation plausible. LncRNA promoters identified by H3K4Me3 show conservation equivalent to protein-coding gene promoters, suggesting primary structure conservation in critical functional elements of lncRNA genes (Guttman et al., 2009). In rodents, there is stronger negative selection on promoters of lncRNAs vs conserved vs genus-specific transcription, indicating that promoter conservation and primary sequence conservation may be positively correlated in lncRNA genes (Kutter et al., 2012). However, comprehensive comparisons of lncRNA promoter and exon conservation genomewide in other lineages have still not been performed. Reverse genetics of the human lncRNAome has yielded a conserved lncRNA, CCAT, which overlaps a colon cancer susceptibility SNP, regulates MYC, and causes a two-fold difference in invasiveness and a four-fold difference in liver metastasis in a mouse colon cancer model (Ling et al., 2013).

While sequence conservation is a hallmark of functional constraint, also known as negative or purifying selection, rapid responses to selective pressures may result in accelerated rates of genetic substitution, also called positive selection, which can also be an indicator of function (Pollard et al., 2006). HAR1 was identified as the most rapidly evolving region of the human genome since the human-chimpanzee common ancestor in a genomewide discovery study of Human-Accelerated Regions, and centers on a shared exonic overlap of the sense and antisense overlapping lncRNA genes HAR1F and HAR1R. HAR1F is co-localized in the embryonic brain with Reelin, which regulates cortical development (Pollard et al., 2006). HAR1 dysregulation has been implicated in Huntington’s disease (Lipovich et al., 2010). HAR1R is up-regulated after the period of cortical development and is thought to regulate HAR1F by antisense inhibition (Pollard et al., 2006). This region, containing a sense-antisense pair of lncRNAs, has evolved in temporal correlation with humanizing traits, highlighting the connection between lncRNA evolution and human-specific phenotypes.

### PSEUDOGENE EVOLUTION: RECENT DISPERSAL

Gencode estimates that there are approximately 14,000 pseudogenes in the human genome (Pei et al., 2012). There is strong quantitative evidence indicating that pseudogenes sharply increase in abundance as speciation occurs and then gradually reduce in frequency (Li et al., 2013). The conclusion that pseudogenes are enabling factors in speciation has been bolstered by the sequencing of primate genomes which showed that regional duplications account for a 2.5% difference in genomic sequence between humans and chimps (Marques-Bonet et al., 2009). Since segmental duplications compose approximately 5% of the genome’s total span, a relatively large proportion of the human genome differs from other primates’ genomes in these regions.

Genes within segmental duplications experience increased copy number variation and decreased selective pressure, increasing the rate of generation of genes with novel functions, as well as pseudogenes (Zhang et al., 2010). The redundancy generated by segmental duplications allows pseudogenes the possibly of gaining new function without being detrimental (Kaessmann, 2010). This mechanism also leads pseudogenes to serve non-adaptive roles, as do lncRNAs, by increasing the reservoir of genetic material that can become a substrate for selection.

Selection against a gene product can lead to loss of function alleles. In CCR5, the homozygous loss of function allele is correlated with protection from HIV-1 and reduced risk of atherosclerosis (Zhang et al., 2010). It is speculated that the deletion appeared in ancestral human populations as a consequence of infection with another pathogen that was detrimental to individuals possessing the full-length functional receptor. The CCR5 gene could become completely pseudogenized if environmental conditions, such as infection with specific pathogens, continue to select against the full-length allele. Although CCR5 is a prominent polymorphism, it is not yet a pseudogene.

In mammals, the lncRNA Xist controls X-inactivation, and is an example of complete pseudogenization. Xist is derived from proto-xist, which was a ubiquitous protein-coding gene before the eutherians and marsupials split (Duret et al., 2006). The Xist locus has three lncRNAs which regulate Xist. Jpx, which is a lncRNA that up regulates Xist by binding the CTCF zinc finger protein (Sun et al., 2013). This lncRNA encoding gene originates form the Uspl gene which was protein-coding in the common ancestor of mammals and avians and is still protein-coding in the domestic chicken (Romito and Rougeulle, 2011). Tsix, which is the antisense transcript of Xist, epigenetically modifies the Xist locus inactivating transcription (Lee et al., 1999). Xist itself is derived from Lnx3, a former protein-coding gene which was protein-coding at least during the time when the common ancestor of marsupials and placental mammals existed (Romito and Rougeulle, 2011). Xite, which is another lncRNA regulator of the Xist locus regulates Tsix (Kung et al., 2013). All three of these lncRNAs originate from protein-coding genes, highlighting a crucial case for the emergence and maintenance of novel non-coding functions from protein-coding genes.

## lncRNA TRANSCRIPTION REGULATING PSEUDOGENES

In this review, we bridge the formerly disparate topics of lncRNA and pseudogene function. We posit that lncRNA transcription is an under-appreciated mechanism of regulating pseudogenes and, hence, the pseudogenes’ downstream effects on their parental genes. Mammalian genomes contain many complex loci. Cis-antisense loci are defined as encompassing pairs of genes which share exonic regions antisense to each other. These loci normally have only one gene which yields a protein product indicating that the non-coding gene may play a role in antisense regulation. Antisense RNA transcription is thought to happen at approximately 20–40% of protein-coding genes; two protein-coding genes, one coding and one non-coding gene, or two non-coding genes may comprise an antisense pair (Chen et al., 2004; Veeramachaneni et al., 2004; Engström et al., 2006). Antisense transcription can also happen at pseudogene, rather than gene, loci; antisense transcription of pseudogenes may be negatively correlated with sense transcription of the pseudogene (Lipovich et al., 2006), a mechanism that affects the regulation of the Oct4 gene by an antisense lncRNA of the Oct4 pseudogene (Hawkins and Morris, 2010). The evolutionary conservation of antisense lncRNAs, and of gene structures at antisense overlaps, in human complex loci is poor outside of primates (Wood et al., 2013); hence, antisense transcripts could play an important role in lineage-specific gene regulation (Lipovich et al., 2006). However, the complexity of genomic-sequence, gene-structure, and transcriptional-orientation conservation (Wood et al., 2013) suggests that local sequence divergence should be re-evaluated from a global continuous primary sequence conservation perspective.

PTEN is a tumor suppressor gene which exemplifies sense and antisense regulatory targeting by a transcribed pseudogene (PTENpg1). PTEN is a negative regulator of the PI3K-AKT pathway and is involved in cell cycle regulation as well as apoptosis. Expression of PTENpg1 leads to the production of three transcripts, two of which are antisense to PTEN. One antisense transcript acts through binding chromatin remodeling complexes which alter H3K27me3 prevalence at the PTEN parental gene promoter (Johnsson et al., 2013). The other antisense transcript is needed to stabilize the PTENpg1 sense transcript, which lacks a poly-A tail. The sense transcript is positively correlated with PTEN activity, consistent with a mechanism where the sense pseudogene transcript works as a sink for microRNA that would otherwise bind the PTEN transcript and deactivate it (Poliseno et al., 2010). PTENpg1 antisense transcription alters doxorubicin sensitivity of cancer cells, a clinically actionable phenotype. These counteracting mechanisms illustrate the importance and complexity of pseudogene-and-lncRNA-mediated regulation, underscoring that important phenotypic effects can result even if the magnitude of the parental gene’s expression change is modest. Nevertheless, the still-emerging lncRNA-pseudogene regulation field is marked by a paucity of experimentally validated examples, and because the lack of a public negative-results repository makes it difficult to assess how many candidate “PTENpg1-like” loci might have already analyzed and shown to lack cellular phenotypes.

The expansion of high throughput expression studies to include pseudogenes is strongly warranted in order to uncover other regulatory examples of this type (Kalyana-Sundaram et al., 2012), and motivates our contention that an empirical genomewide assessment of sense and antisense transcription at other loci containing pseudogene-lncRNA overlaps, and their impact on the regulation of the pseudogenes’ parental genes in human health and disease, is indispensable. The availability of discrete lncRNA and pseudogene catalogs, from resources including Gencode, makes such an assessment feasible. Multispecies genome and transcriptome repositories are expected to empower our understanding of the evolutionary novelties responsible for coordinated pseudogene and lncRNA-mediated gene regulation uniquely in the primate lineage.

## CONCLUSION

Here, we argue that synergistic gene regulation by pseudogenes and lncRNAs needs to be considered as a novel regulatory mechanism. We canvassed the literature for evidence supporting lncRNA regulation of pseudogenes as well as transcription of pseudogenes into lncRNA, and we conclude that there is potential for these events to occur together across numerous genomic loci. Expression of lncRNA regulates pseudogene loci and hence leads to effects which propagate through the genome to the pseudogenes’ parental genes. Despite this evidence for lncRNA and pseudogene function on a case by case basis, there is still a generalized dearth of expressed pseudogene functional support, particularly within the genomewide context of pseudogene overlaps with lncRNA genes.

LncRNA genes and transcribed pseudogenes are typically identified by using CAGE, mRNA, EST databases, and gene identification signature paired end tags. Short-tag mapping at pseudogene loci can be ambiguous, although hybrid approaches with machine learning are being applied to facilitate transcribed-pseudogene discovery (Valdes and Capobianco, 2014). Hence, in order to improve upon these techniques, full-length expression data must be generated. While cDNAs and ESTs that clearly map to the pseudogene rather than to the parental gene have provided reliable windows into pseudogene transcription, the use of third-generation sequencing techniques including Pacific Biosciences and Oxford Nanopore for transcriptome characterization would therefore greatly improve the accuracy of expression profiles.

LncRNAs can carry out both gene inhibition and gene activation, and prior studies indicated that the choice of synergistic, vs. reciprocal gene regulation is complex and depends on diverse factors such as transcriptional orientation of lncRNA and coding genes at each complex locus as well as developmental and epigenetic states. We posit that lncRNAs overlapping with pseudogenes are also a potential contributor to both the magnitude and the directionality of this regulation. Therefore, new data sets should also address the orientation of pseudogenes relative to their cognate lncRNA transcripts. Gencode provides strand specific transcript models that are capable of adding this type of depth to future studies. The raw data needed to generate these genomewide overlaps of lncRNAs and pseudogenes is currently available through the UCSC Genome Browser, the ENCODE Consortium, and other lncRNA and pseudogene reference sets. The confidence of lncRNA-pseudogene overlaps can be bolstered by sense and antisense EST and mRNA transcriptional evidence at the overlap loci, which is also available. The significance of these overlaps in regard to regulation of genes by pseudogenes and lncRNAs, including sense and antisense lncRNA transcripts from pseudogene loci, should be more completely explored. In view of pseudogenes’ and lncRNA’s exaptive properties, as well as the accumulating evidence indicative of recent evolution of pseudogenes and lncRNAs, their future study will undoubtedly lead to an enhanced understanding of the roles that pseudogenes and ncRNAs play in evolution. Insights would address the recent emergence of lncRNA-mediated organismal functions that are exerted through the transcription of lncRNAs from, and/or antisense to, pseudogenes. In particular, these new datasets are poised to provide detailed insights into the relevance of newly arising lncRNAs and pseudogenes to primate speciation and lineage-specific phenotypes, with direct functional links to recent evolutionary events that have influenced human susceptibility to cancer and other diseases. We posit that numerous additional examples of joint lncRNA- and pseudogene-driven regulation of protein-coding genes are waiting to be discovered in post-genomic datasets. The rapidly growing datasets of significantly disease-associated SNPs from Genome-Wide Association Studies, a resource that has empowered the realization that most trait-associated loci are not protein-coding (Kellis et al., 2014), are likely to provide a goldmine of intrapseudogenic and lncRNA exonic disease-associated SNPs which can then pave the way to functional studies for decades to come.

### Conflict of Interest Statement

The authors declare that the research was conducted in the absence of any commercial or financial relationships that could be construed as a potential conflict of interest.
